# Tumor cells versus host immune cells: whose PD-L1 contributes to PD-1/PD-L1 blockade mediated cancer immunotherapy?

**DOI:** 10.1186/s13578-018-0232-4

**Published:** 2018-05-02

**Authors:** Fei Tang, Pan Zheng

**Affiliations:** Division of Immunotherapy, Institute of Human Virology and Department of Surgery, University of Maryland School of Medicine, 725 W Lombard Street, Baltimore, MD 21201 USA

**Keywords:** PD-L1, PD-1/PD-L1 blockade, Cancer immunotherapy, Host immune cells, Immune evasion, Immune therapeutic effect

## Abstract

Antibody blockade of the PD-1/PD-L1 pathway has elicited durable antitumor responses in the therapy of a broad spectrum of cancers. PD-L1 is constitutively expressed in certain tumors and host immune cells, and its expression can be induced or maintained by many factors. The expression of PD-L1 on tumor tissues has been reported to be positively correlated with the efficacy of anti-PD-1/PD-L1 therapy in patients. However, multiple clinical trials indicate that patients with PD-L1-negative tumors also respond to this blockade therapy, which suggests the potential contribution of PD-L1 from host immune cells. Recently, six articles independently evaluated and verified the contributions of PD-L1 from tumor versus non-tumor cells in various mouse tumor models. These studies confirmed that PD-L1 on either tumor cells or host immune cells contributes to tumor escape, and the relative contributions of PD-L1 on these cells seem to be context-dependent. While both tumor- and host-derived PD-L1 can play critical roles in immune suppression, differences in tumor immunogenicity appear to underlie their relative importance. Notably, these reports highlight the essential roles of PD-L1 from host myeloid cells in negatively regulating T cell activation and limiting T cell trafficking. Therefore, comprehensive evaluating the global PD-L1 expression, rather than monitoring PD-L1 expression on tumor cells alone, should be a more accurate way for predicting responses in PD-1/PD-L1 blockade therapy in cancer patients.

## Background

**Fig. 1:**
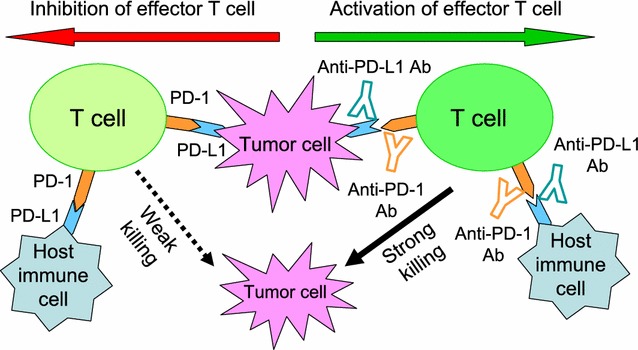
PD-L1 on either tumor cells or host immune cells is proposed to function in preventing T cell-mediated tumor killing. PD-1 is highly expressed in exhausted effector T cells. PD-L1 is constitutively expressed in some tumors and host immune cells, and its expression can be induced or maintained by many factors. PD-1-PD-L1 interaction drives T cell dysfunction, which results in a weaker tumor killing ability in effector T cells. Therefore, anti-PD-1/PD-L1 antibodies-mediated specific blockade of the PD-1/PD-L1 pathway can enhance antitumor immunity

Antibody blockade of the programmed death-1 receptor/programmed death-ligand 1(PD-1/PD-L1) signaling pathway has shown unprecedented durable therapeutic responses in patients with a variety of cancers. Accumulating studies in animal models and clinical trials have contributed to our current understanding of mechanisms underlying the efficacy of PD-1/PD-L1 pathway blockade in cancer immunotherapy. Since PD-L1 on tumor cells plays an important role in preventing T cell-mediated killing, beneficial outcome of PD-1/PD-L1 blockade therapy has been correlated with PD-L1 expression on tumor cells [1]. Besides tumor cells, various types of host cells also constitutively express PD-L1, and PD-L1 can be upregulated on many cells when stimulated by inflammatory cytokines like interferons (IFNs). Moreover, multiple clinical trials indicate that patients with PD-L1-negative tumors also respond to this blockade therapy [2], suggesting the potential contribution of PD-L1 from host immune cells. However, the dynamic change of PD-L1 expression within the tumor microenvironment has made it difficult to identify the specific PD-L1-expressing cells that contribute to a tumor’s immune evasion (Fig. 1).

Elucidation on the contributions of tumor cells and host immune cells-derived PD-L1 has important clinical implications as PD-L1 expression may predict the sensitivity of anti-PD-1/PD-L1 immunotherapy in cancer patients. Within 1 year from early of 2017, six independent research groups published papers in high impact journals and explained their points of view on the contributions of PD-L1 expressed from relevant cells [3–8]. Mouse tumor models involving multiple tumor cell lines and mice with various genetic backgrounds were used in these studies (Table 1). All the researchers investigated the role of PD-L1 expressed on different cell types within the tumor-microenvironment, and these studies greatly complement our understanding of molecular and cellular mechanisms that account for the clinical efficacy of PD-L1 and PD-1 blockade. In the following, we would like to highlight the main discoveries and points of view from the authors in chronological order of publication of these articles.

**Table 1:** Summary on the major tumor cell lines, mouse models and points of view from 6 independent studies

Authors	Journal	Major tumor cells used	Major mouse models used	Proposed source(s) of PD-L1 contributed to tumor evasion
Noguchi et al. [3]	*Cancer Immunology Research*	MCA-induced sarcoma T3 and T9; T3ΔPDL1 clones; T9-PD-L1^ovr^ clone; T9-PD-L1^phy^ clone;	129S6 WT and Rag2^−/−^ mice	Both tumors and host immune cells (particularly tumor associated macrophages)
Lau et al. [4]	*Nature Communications*	Colon tumor MC38 and CT26; PD-L1-KO/inducible MC38/CT26 clones;	BALB/c; C57BL/6; Rag2^−/−^; PD-L1^−/−^ mice	Disparate cellular sources, including tumor cells, myeloid or other immune cells
Kleinovink et al. [5]	*OncoImmunology*	MC38 and CT26; PD-L1-KO MC38/CT26 clones	C57BL/6; BALB/c mice	Both malignant cells and immune cells
Juneja et al. [6]	*The Journal of Experimental Medicine*	MC38; melanoma B16.F10; BRAF.PTEN; PD-L1-KO MC38/BRAF.PTEN clones	C57BL/6; PD-1^−/−^; PD-L1^−/−^; PD-L1^−/−^PD-L2^−/−^ mice	Context-dependent; For MC38 model, PD-L1 on tumors; For BRAF.PTEN and B16.F10 + Gvax models, PD-L1 on non-tumor cells
Tang et al. [7]	*The Journal of Clinical Investigation*	MC38, B lymphoma A20; T lymphoma E.G7; PD-L1-KO MC38/A20 clones	C57BL/6; BALB/c; Rag1^−/−^; CD11b-DTR; NSG; PD-L1^−/−^ mice	The contribution of PD-L1 on tumor cells is largely dispensable; PD-L1 on host myeloid cells is essential
Lin et al. [8]	*The Journal of Clinical Investigation*	MC38; B16-F10; lung cancer LLC; ovarian cancer ID8; PD-L1-KO/over-expression MC38/ID8/B16-F10 clones;	C57BL/6; NSG; Rag1^−/−^; PD-L1^−/−^; PD-1^−/−^ mice	PD-L1 on tumor cells does not contribute to PD-L1 blockade efficacy; PD-L1 on host DCs and macrophages predicts clinical efficacy of PD-L1/PD-1 blockade

T3ΔPDL1; T3 cells with deficiency of PD-L1; T9-PD-L1^ovr^: T9 cells with over-expression of PD-L1; T9-PD-L1^phy^: T9 cells with physiological levels of PD-L1 expression; KO: knock out; DTR, diphtheria toxin receptor; NSG: NOD (non-obese diabetic) SCID (severe combined immunodeficiency) gamma mice; B16.F10 + Gvax, B16.F10 melanoma combined with GVAX

## Major discoveries and points of view on roles of tumor- and host-derived PD-L1 in tumor immune evasion

Noguchi et al. [3] generated multiple MCA (methylcholanthrene)-induced sarcoma cell lines in their study. T3 is one of the sarcoma cell lines with low immunogenicity, and is sensitive to PD-1/PD-L1 blockade therapy. The authors used multiple T3-based sarcoma lines lacking PD-L1 (T3ΔPDL1), WT and Rag2^−/−^ mice to test whether PD-L1 expression on tumor cells was required for tumor immune escape. While growing T3ΔPDL1 tumors were observed in all of the Rag2^−/−^ mice, the majority of inoculated T3ΔPDL1 clones were spontaneously rejected in syngeneic WT mice. Furthermore, T3ΔPDL1 cells with enforced expression of PD-L1 regained the capacity to form progressively growing tumors in WT mice. These facts revealed that PD-L1 expression on T3 sarcoma cells was functional in suppressing antitumor immunity in the model. In addition, Noguchi et al. [3] observed that the number of WT mice with progressively growing T3ΔPDL1 tumors increased if more cells were used for initial tumor inoculation. Moreover, anti–PD-L1 treatments induced tumor rejection in these mice, suggesting PD-L1 expression on host cells also participated in preventing immune elimination of PD-L1-deficient sarcoma cells.

Lau et al. [4] used genetic deletion of PD-L1 in MC38 and CT26 colorectal tumor cells and host mice to study T cell inhibition by PD-L1 therapy. Knocking down of PD-L1 in both tumors spontaneously resulted in tumor growth in their models, whereas therapeutic PD-L1 blockade augmented anti-tumor T cell responses and further extended survival, suggesting that PD-L1 expression by both the tumor and host plays distinct, partial roles in regulating anti-tumor immunity. They also compared tumor growth in MC38 models with PD-L1 deficiency on the tumor, the host, and both compartments. Despite PD-L1 loss in the tumor or host compartment led to tumor regressions, a subset of tumors achieved sustained growth. However, when neither the tumor nor the host cells expressed PD-L1, they observed the highest rate of tumor regressions with near complete prevention of tumor escape. These results imply that PD-L1 from tumor and host compartment works in concert to dampen the antitumor immune response. Consistently, their gene expression analyses showed the strongest enrichment for T cell immunity-related genes when PD-L1 was lacking on both tumor cells and host cells.

Kleinovink et al. [5] have also described a non-redundant role of PD-L1 expression on tumor cells and host cells for mediating immune suppression in the widely used MC38 and CT26 tumor models. PD-L1 knockout by CRISPR-Cas9 technology in both cell types rendered tumors slower growth than their WT counterpart cells. Moreover, PD-1 or PD-L1 blockade with therapeutical antibodies still effectively eradiated the outgrowing tumors, which suggests an additional role for PD-L1 on host-derived immune cells within the tumor microenvironment. The authors also performed antibody-mediated T cell depletion experiments in mice bearing PD-L1-deficiency MC38 tumors. Their study emphasizes the crucial role of CD8^+^ T cells for the antitumor effects of PD-L1 antibody therapy.

Juneja et al. [6] first tested the roles of PD-L1 on tumor cells and non-tumor cells through implanting MC38 tumors or B16 melanoma cells into WT and PD-L1/PD-L2-deficiency mice. In the PD-L1^−/−^ PD-L2^−/−^ mice, PD-L1 expression on the tumor cells is the only source of ligands for PD-1.They found that MC38 tumor growth was similarly robust in PD-L1^−/−^ PD-L2^−/−^ and WT mice, which indicates that engagement of PD-1 by PD-L1 on tumor cells alone is sufficient to suppress antitumor immunity to MC38 tumors. Consistently, administration of PD-L1-blocking antibody to MC38 tumor-bearing PD-L1^−/−^ mice resulted in tumor clearance in the majority of mice. Juneja et al. [6] also used two tumor models that are only moderately sensitive to PD-1 blockade, BRAF.PTEN melanoma and B16.F10 melanoma combined with GVAX, to test the relative importance of PD-L1 on tumor cells versus non-tumor cells. Unlike MC38 tumors, the growth of these melanoma cells with lower immunogenicity was delayed in PD-L1^−/−^ mice compared with WT mice. This suggests that PD-L1 expression on non-tumor cells in WT mice plays a nonnegligible role in inhibiting antitumor immunity to melanoma tumors.

Tang et al. [7] used MC38 tumor and A20 (B lymphoma) tumor cells, and PD-L1 knockout mice, BM transplantation chimera mice, CD11b-DTR (diphtheria toxin receptor) mice, as well as various depletion antibodies, to address the contribution of PD-L1 from relevant cells in checkpoint blockade therapy. Their data suggest that PD-L1 on tumor cells is not essential for the response to PD-L1 blockade in their models, and myeloid cells derived PD-L1 is sufficient to limit immune response. Using real-time imaging in whole tumor tissues, they observed that anti-PD-L1 antibody accumulated in tumor tissues, regardless of the status of PD-L1 expression on tumor cells. They confirmed that T cells are essential in anti-PD-L1-mediated tumor regression, and effective lymphocyte trafficking to tumor tissues is required for overall responses. In particular, via CD11b-DTR/PD-L1^−/−^ mixed bone marrow chimera mice model, they elegantly demonstrated that blocking PD-L1 on CD11b^+^ myeloid cells is indispensable for effective antitumor immunity in PD-L1 blockade therapy.

Lin et al. [8] used mice with varying immune repertoires, including Rag1^−/−^, NSG, PD-L1^−/−^ and PD-1^−/−^ mice, for studying PD-L1 and PD-1 signaling blockade in MC38, ID8 (ovarian cancer), B16-F10 (melanoma), and LLC (lung cancer) tumor models. First, they verified that host immunity determines anti-PD-L1-induced tumor immunity. While WT mice bearing various tumors had effective response to PD-L1 blockade, anti-PD-L1 treatment had no antitumor effect in NSG and Rag1^−/−^ mice. Their further experiments indicate that anti-PD-L1 treatment reduced tumor growth in mice bearing PD-L1-deficient MC38, ID8 and B16-F10 tumors, which implies that host- but not tumor-derived PD-L1, is indispensable for the therapeutic efficacy of anti-PD-L1 treatment. Mechanistically, the authors experimentally proved that anti-PD-L1 treatment activates T cells in tumor and draining lymph nodes. They demonstrated that tumor-associated APCs (antigen-presenting cells), as the major PD-L1^+^ immune cells, are the major immune targets of anti-PD-L1 therapy. Additionally, they observed a well correlation between expression of PD-L1 on dendritic cells (DCs)/macrophages and the efficacy of treatments with either anti–PD-1 alone or in combination with anti-CTLA-4 in ovarian cancer and melanoma patients. They concluded that the host immune system is indispensable for PD-1/PD-L1 blockade therapy, and the host DCs and macrophages-derived PD-L1, rather than cancer cell-intrinsic PD-L1, predominantly accounts for the blockade therapeutical efficacy.

## Multiple experimental approaches with genetic deletion of PD-L1 on tumor cells or the host cells

**Fig. 2:**
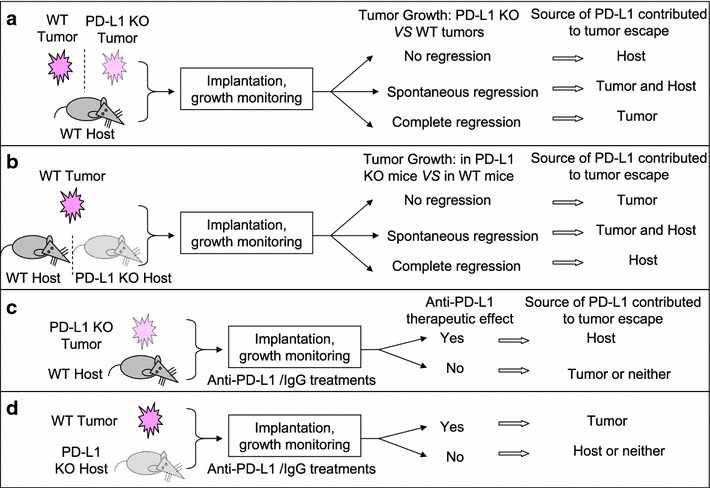
Multiple experimental approaches through genetic deletion of PD-L1 on tumor cells or the hosts can be employed to elucidate the contribution of PD-L1 in mediating tumor evasion.** a** PD-L1-sufficient or PD-L1 knock out (KO) tumor cells are inoculated into WT immunocompetent hosts, and the source of PD-L1 contributed to tumor escape is determined by the regression of PD-L1-deficient tumors.** b** WT tumors are inoculated into WT or PD-L1-deficient hosts, and the source of PD-L1 contributed to tumor escape is determined by the tumor regression in PD-L1 KO hosts.** c**,** d** PD-L1-KO tumors are inoculated into WT host (**c**), or WT tumors are inoculated into PD-L1 KO hosts (**d**), and the source of PD-L1 contributed to tumor escape is determined by the therapeutic effects of anti-PD-L1 antibody

From multiple angels and using various techniques and pre-clinical tumor models, these studies reveal that PD-L1 on tumor cells and host cells is involved in tumor immune evasion. All the studies employed gene silencing technologies to knock out of PD-L1 in tumors, and these tumors were inoculated to WT immune competent hosts to test the roles of PD-L1 in tumor immune escape (Fig. 2a). While reports from Noguchi et al. (T3ΔPDL1 tumors), Lau et al. (MC38 tumors), Kleinovink et al. (MC38 and CT26 tumors), and Juneja et al. (MC38 and BRAF.PTEN tumors) showed spontaneous regression or slow growth of tumors [3–6], Tang et al. (MC38 and A20 tumors) did not observe significant growth differences in tumors with loss of PD-L1 expression [7]. Rather than directly comparing the growth of PD-L1^−/−^ and PD-L1^+/+^ tumors in WT host, Lin et al. demonstrated the critical role of host-derived PD-L1 by treating the host with anti-PD-L1 antibody, which resulted in significant tumor regression [8]. Both Tang et al. and Lin et al. are inclined to underscore the essential roles of PD-L1 from host myeloid cells in mediating PD-1/PD-L1 blockade therapeutic effects, whereas others’ studies emphasize that PD-L1 from tumor and host compartment works in concert. Although Juneja et al. demonstrated evidence of PD-L1 on MC38 cells in inhibiting CD8^+^ T cell cytotoxicity and suppressing antitumor immunity in their model [6], Tang et al. and Lin et al. concluded that PD-L1 on tumor cells is largely dispensable for the response to checkpoint blockade [7, 8]. The discrepancy among these studies is probably due to different experimental setups, for examples, mouse strains, reagents, and the amounts of initially inoculated tumors. Indeed, Noguchi et al. observed that when WT mice were challenged with increasing numbers of T3ΔPDL1 tumor cells, the number of mice with progressively growing tumors increased [3]. This suggests that the initial amount of challenged tumor antigens matters as it can affect the ability of host in immunologically elimination of malignancies.

Most of these studies implanted PD-L1-sufficient tumors to PD-L1 knock out hosts to test the contribution of PD-L1 in suppressing the antitumor T cell responses (Fig. 2b). While Lau et al. observed spontaneous regression of MC38 tumors in PD-L1 KO hosts [4], Juneja et al. noticed that MC38 tumor growth was similarly robust in PD-L1^−/−^PD-L2^−/−^ and WT mice [6]. This discrepancy, again, can be explained by different experimental settings. In addition, Juneja et al. proposed model-dependent role of PD-L1 on tumor cells versus non-tumor cells, as they found that in BRAF.PTEN melanoma and B16.F10 + GVAX models, tumors had delayed growth in PD-L1^−/−^ mice compared with WT mice [6]. In addition, the source of PD-L1 contributed to tumor escape can be determined by therapeutic effects of anti-PD-L1 antibody (Fig. 2c, d). For example, Tang et al. and Lin et al. evaluated the contribution of tumor-derived PD-L1 by treating tumor bearing PD-L1 KO host with anti-PD-L1 antibodies [7, 8]. However, the therapeutic effects of PD-L1 blockade were not observed in tested tumor models (MC38, ID8 and B16-F10 tumors) of these two studies. Therefore, they tend to advocate that host expression of PD-L1 determines efficacy of PD-L1 pathway blockade-mediated tumor regression. Even though the contribution of tumor cells-derived PD-L1 appears dispensable, their work does not rule out the possibility that tumor-derived PD-L1 could play important roles during the early phase of tumor establishment or when PD-L1 is constitutively highly expressed on tumor cells, as they noted [7, 8].

Besides, functional consequences of different levels of PD-L1 expression on tumors were assessed from Noguchi et al. by comparing physiological level and over-expressed level of PD-L1. They found that abnormally high expression of PD-L1, but not levels of PD-L1 expression that can be induced on tumor cells under physiologic conditions, is required to prevent immune elimination of highly immunogenic unedited MCA sarcoma cells that express strong neoantigens [3]. Their work demonstrates the inverse relationship between tumor antigenicity and the capacity of PD-L1 to promote tumor escape. Therefore, to some extent, tumor immunogenicity, as proposed by Juneja et al., seems to underlie the relative importance of tumor- and host-derived PD-L1 [6]. In addition, by mixed competition assays, both Juneja et al. and Lau et al. demonstrated that tumor PD-L1 conferred a selective advantage in proliferation, as PD-L1-sufficient MC38 cells outcompeted PD-L1-deleted MC38 cells in vivo. This finding underlies the significant role of PD-L1 as a molecular shield on tumor cells to protect them from elimination within the tumor microenvironment [4, 6]. Since simultaneous deletion of PD-L1 from both tumor and host compartments led to most profound frequency of tumor regressions, this unique work from Lau et al. argues for a non-redundant contribution of PD-L1 from disparate cellular sources [4].

Collectively, these findings demonstrate that both tumor- and host-derived PD-L1 can play a critical role in inhibiting antitumor immunity, and the relative contribution of tumor- or host-derived PD-L1 is context-dependent. All the studies confirmed the role of PD-L1, either from tumors or host cells, in suppressing T cell functions, as evidenced by changes in cytotoxicity of effector T cells and the secretion of effector cytokines like IFNγ. However, these articles have different focuses on revealing mechanisms of PD-L1 in suppressing anti-tumor immunity. Noguchi et al. conclude that tumor-associated macrophages (TAMs) are the major host cell type that contributes PD-L1 in the sarcoma tumor model both quantitatively and temporally. In their study, while in vivo up-regulation of PD-L1 on T3 tumor cells was in a transient and time-dependent manner, PD-L1 expression on TAMs was retained for long period of time, and can be induced by CD4^+^ T cells dependent cell-extrinsic pathways [3]. Lau et al. performed RNA profiling and described several alternative immune escape mechanisms in outgrowing PD-L1^−/−^ tumors, including reduced MHC-I expression and increased PD-L2 expression [4]. Juneja et al. showed that PD-L1 on MC38 tumor cells is sufficient to directly suppress activated tumor-infiltrated antigen-specific CD8^+^ cytotoxic T lymphocytes (CTLs), and is dominant in suppression of antitumor immunity in their mouse model [6]. Tang et al. highlighted the roles of CD11b^+^PD-L1^+^ myeloid cells and enhancing effective T cell trafficking in contributing to the efficacy of PD-L1 blockade therapy [7]. The work from Lin et al. concentrated on evaluating therapeutic efficacy of PD-L1 and PD-1 blockade in both mice and human, with a focus on functional PD-L1 expression in DCs and macrophages in the tumor microenvironment and draining lymph nodes [8].

## Concluding remarks

**Fig. 3:**
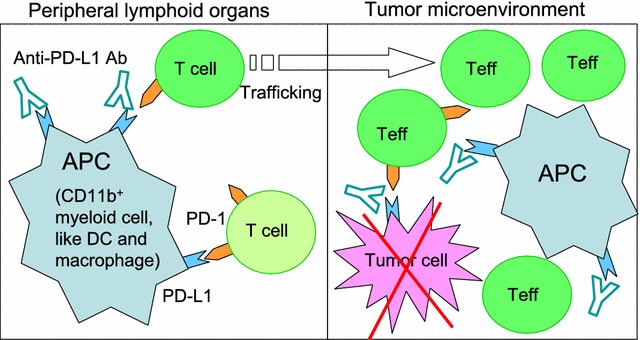
A comprehensive understanding of PD-L1 on both tumor cells and immune cells in working concretely to contribute to anti-PD-L1 blockade mediated therapeutic effects. PD-L1 expressed on either tumors or host immune cells contributes to the inhibition of T cell activation, which can be released through blocking PD-L1 signaling by antibodies. During antitumor immune responses, antigen-presenting cells (APCs, especially dentritic cells and macrophages) uptake tumor antigens and activate T cells inside tumors. PD-L1 blockade on both APCs and tumor cells within the tumor microenvironment enhances T cell activation. APCs with up-taken tumor antigens migrate from the tumor tissues to the peripheral lymphoid organs to activate T cells outside tumors. Peripherally activated tumor-specific effector T cells infiltrate into the tumor sites and immunologically eliminate tumors. Peripheral PD-L1 signaling in draining lymph nodes may significantly hamper the antitumor immune responses by limiting effector T cell trafficking. Thus, PD-L1 blockades both inside and outside tumors may jointly promote host immunity to achieve effective therapy. Therefore, PD-L1 expression on either tumors or immune cells could be predictive of sensitivity to therapeutic agents targeting the PD-1/PD-L1 axis. Teff, effector T cell

Taking together, these independent studies largely complement and validate each other. Their work extends the mechanisms of action of PD-1/PD-L1 blockade therapy into the tumor microenvironment, and largely supports the concept that PD-L1 acts as a molecular shield on both tumor cells and host immune cells to prevent tumors from cytolysis by T cells. PD-L1 expressed on cancerous cells is not exclusively responsible for the therapeutic effect of PD-1/PD-L1 checkpoint blockade, whereas PD-L1 on both malignant cells and immune cells works concretely to functionally modulate the CTLs in the tumor microenvironment (Fig. 3). Therefore, PD-L1 from both sources could be predictive of sensitivity to therapeutic agents targeting the PD-1/PD-L1 axis. These studies clearly suggest that a comprehensive evaluation of total PD-L1 expression in the tumor microenvironment, rather than monitoring PD-L1 expression on tumor cells alone, may represent a more accurate approach for predicting the effects of PD-1/PD-L1 blockade therapy in cancer patients. Recently, we reported that inactivation of mTORC1 (the mammalian target of rapamycin, complex 1) signaling in hematopoietic stem/progenitor cells causes a massive expansion of previously uncharacterized CD11b^+^ PD-L1^+^ innate myelolymphoblastoid effector cells (IMLECs) [9]. It is worth noting that recent work from Tang et al. and Lin et al. elegantly underscores the indispensable and dominant roles of CD11b^+^ PD-L1^+^ myeloid cells in contributing to the efficacy of PD-1/PD-L1 blockade therapy in some tumors [7, 8]. Therefore, for patients being treated with some conventional anticancer drugs such as mTOR inhibitors, potentially induced PD-L1 on host immune cells may be a driving force that cannot be ignored in tumor immune escape. Thus, on the other hand, the work on illustrating the critical roles of PD-L1 on host immune cells might have broad implications for the explanation of anticancer drug resistances in some patients.

## Abbreviations

Ab: antibody
APC: antigen-presenting cell
CRISPR-Cas9: clustered Regularly interspaced short palindromic repeats and CRISPR-associated protein 9
CTL: cytotoxic T lymphocyte
CTLA-4: cytotoxic T-lymphocyte associated protein 4
DC: dendritic cell
DTR: diphtheria toxin receptor
IFN: interferon
KO: knock out
MCA: methylcholanthrene
NSG mice: NOD (non-obese diabetic) SCID (severe combined immunodeficiency) gamma mice
PD-1: programmed death-1
PD-L1: programmed death-ligand 1
TAM: tumor-associated macrophage
Teff: effector T cell
